# Supplementary material for: Ran promotes membrane targeting and stabilization of RhoA to orchestrate ovarian cancer cell invasion
Source: Nat Commun. 2019 Jun 17;10:2666. doi: 10.1038/s41467-019-10570-w (PMC6573066; doi:10.1038/s41467-019-10570-w)

Supplementary Information

Ran promotes membrane targeting and stabilization of RhoA to orchestrate ovarian cancer cell invasion

Zaoui et al.

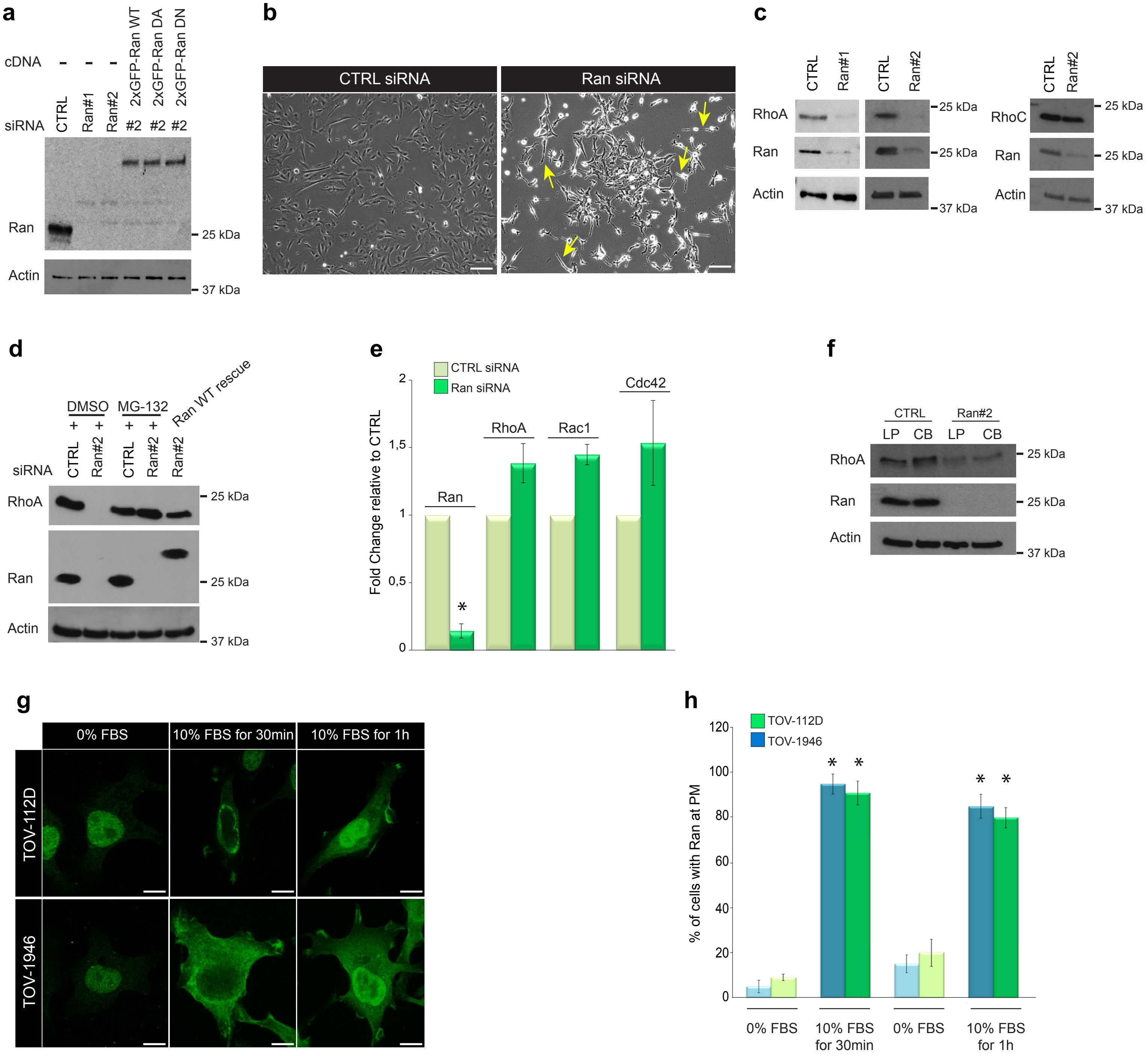

Supplementary Figure 1 Ran GTPase localizes to the plasma membrane and stabilizes RhoA protein in EOC cells. (a) Western blot of Ran knockdown (KD) with siRNA (CTRL, Ran #1 or 2) and rescue levels with different RNAi-resistant 2xGFP constructs of Ran as wild-type (WT), dominant active (DA), and dominant-negative (DN) in TOV-1946 cells. Actin served as a loading control for all blots. (b) Live cell images of TOV-112D cells at 72 hours post-transfection with control or Ran #2 siRNAs. Arrows indicate the tail of Ran-depleted cells. Scale bars, 100 µm. (c) Western blot showing RhoA and RhoC protein expression levels after Ran KD in TOV-1946 cells. (d) Western blot showing RhoA protein level after re-expression of 2xGFP-Ran WT (Ran WT rescue) or treatment for 2 hours with 20 µM MG-132 in TOV-1946 cells transfected with Ran #2 siRNA. (e) Comparison of the fold change in transcripts of RhoA, Rac1 and Cdc42 following Ran KD in TOV-112D were assessed by quantitative RT-PCR with β-actin as an internal control. All values are from three independent experiments. P-values are based on comparisons with CTRL using the *t*-test: *, $P < 0.05$ was considered statistically significant. (f) Cell body (CB) and lamellipodia (LP) of control (CTRL) and Ran KD TOV-1946 cells were fractionated. Equal amounts of proteins were immunoblotted to show RhoA expression in the respective fractions. RhoA was decreased in CB and LP in Ran KD cells but unchanged in CTRL. (g) TOV-112D and TOV-1946 cells were stimulated with FBS for 30 minutes and 1 hour. Cells were fixed, permeabilized, and subjected to immunofluorescence using Ran antibody and visualized by spinning disk microscopy. Scale bars, 10 µm. (h) Percentage of TOV-112D and TOV-1946 cells with Ran at the plasma membrane (PM) was scored. All values are means ± SEM from three independent experiments. P-values are based on comparisons with 0% FBS using the *t*-test: *, $P < 0.05$ was considered statistically significant.

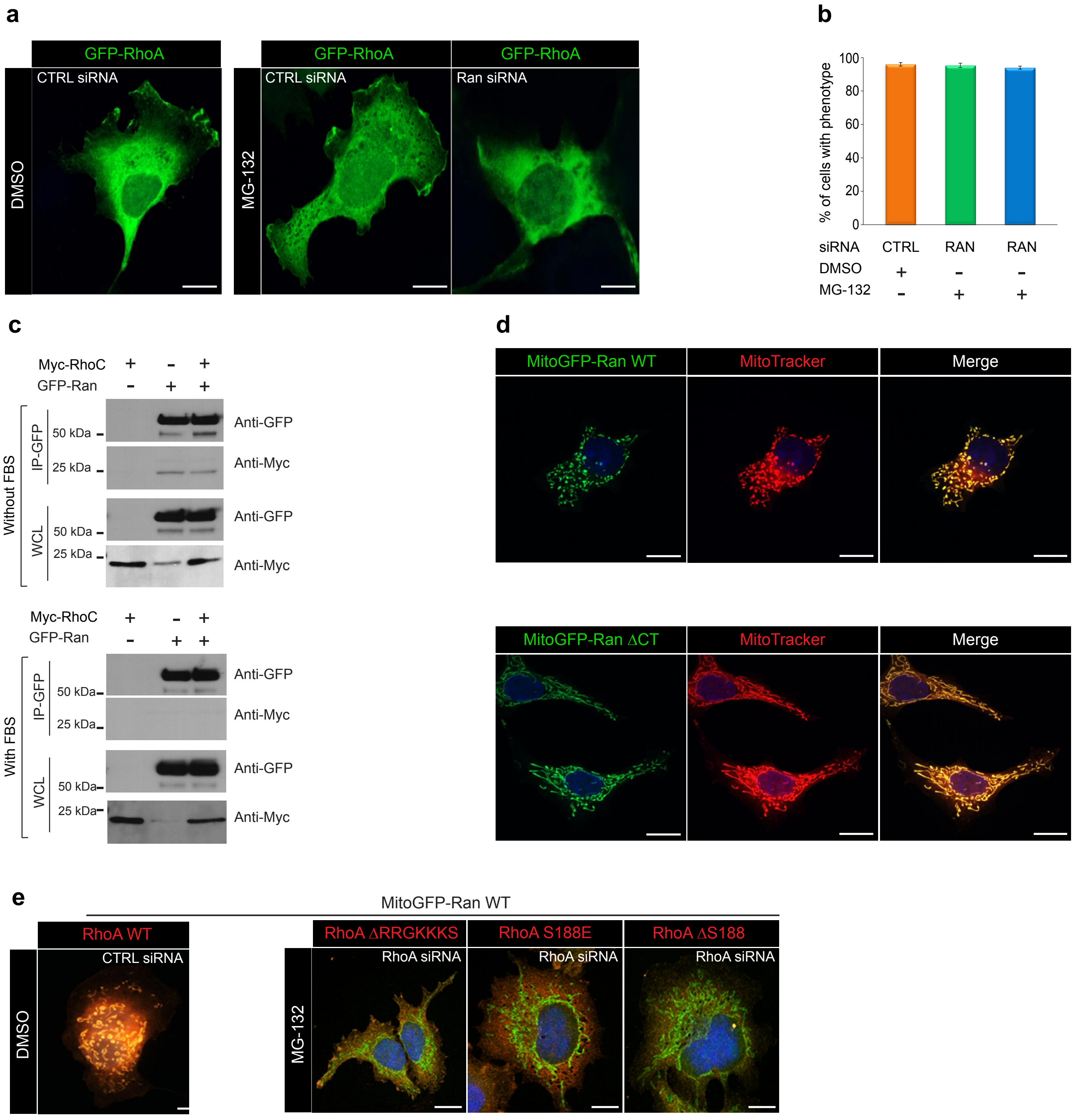

Supplementary Figure 2 Ran GTPase localizes to the plasma membrane and stabilizes RhoA protein. (a) TOV-112D cells expressing GFP-RhoA were co-transfected with CTRL siRNA or Ran and treated with DMSO or 20 μM MG-132 for 2 hours as indicated. Cells were then fixed, permeabilized, and subjected to immunofluorescence and visualized by spinning disk microscopy. Scale bars, 10 μm. (b) Percentage of TOV-112D cells with corresponding phenotype as in (a) for RhoA localization at the PM or not, was scored. (c) TOV-112D cells co-transfected with Myc-RhoC WT and GFP-Ran WT were subjected to immunoprecipitation (IP) by an anti-GFP antibody and western blotted as shown. (d) TOV-112D cells transfected with MitoGFP-Ran WT or MitoGFP-Ran ΔCT (Ran without DEDDDL motif) were incubated for 30 minutes with the mitochondrial dye MitoTracker® to confirm that MitoGFP-Ran WT or MitoGFP-Ran ΔCT (Ran without DEDDDL motif) localized to the mitochondria. (e) TOV-112D cells expressing MitoGFP-Ran WT (green) were co-transfected with CTRL or Ran siRNA and either Myc-RhoA (WT), Myc-RhoA (ΔRRGKKKS), Myc-RhoA (ΔS188) or Myc-RhoA (S188E) mutants. Cells were treated with DMSO or with 20 μM MG-132 for 2 hours, then fixed, permeabilized, and subjected to immunofluorescence using an anti-Myc antibody. Cells were visualized by spinning disk microscopy. Scale bars, 10 μm.

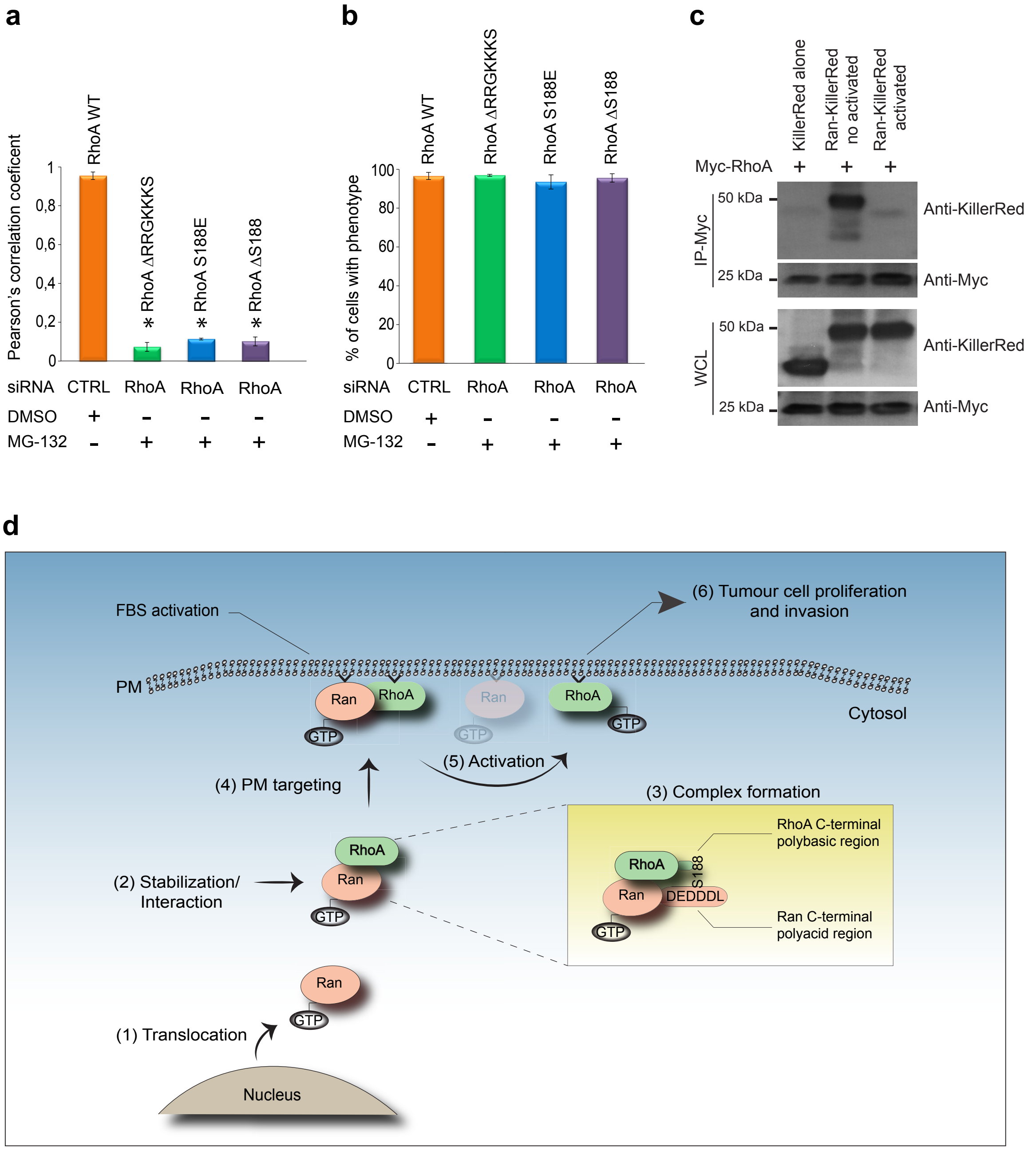

Supplementary Figure 3 The serine 188 residue within the C-terminus domain of RhoA is crucial for functional interaction with Ran protein. (a) Co-localization between MitoGFP-Ran WT and either Myc-RhoA (WT), Myc-RhoA (ΔRRGKKKS), Myc-RhoA (ΔS188) or Myc-RhoA (S188E) mutants was represented as Pearson's correlation coefficient and measured for individual TOV-112D cells treated with DMSO or with 20 μM MG-132 for 2 hours. All values are means ± SEM from three independent experiments. P-values are based on comparisons with CTRL (RhoA WT) using the *t*-test: *, $P < 0.05$ was considered statistically significant. (b) Percentage of TOV-112D cells with corresponding phenotype as in (Supplementary Fig. 2e) for RhoA WT, RhoA ΔRRGKKKS, RhoA ΔS188 or RhoA S188E mutants colocalization or not to the mitochondria, was scored. (c) Immunoprecipitation (IP) of RhoA protein using an anti-Myc antibody was performed in TOV-112D cells co-transfected with Myc-RhoA (WT) and membrane anchored Ran-KillerRed, before and after KillerRed inactivation and western blotted as shown. KillerRed inactivation caused a dissociation of the Ran-RhoA interaction. (d) Schematic model of Ran-RhoA signaling axis on tumour cell proliferation and invasion.

Supplementary Figure 4

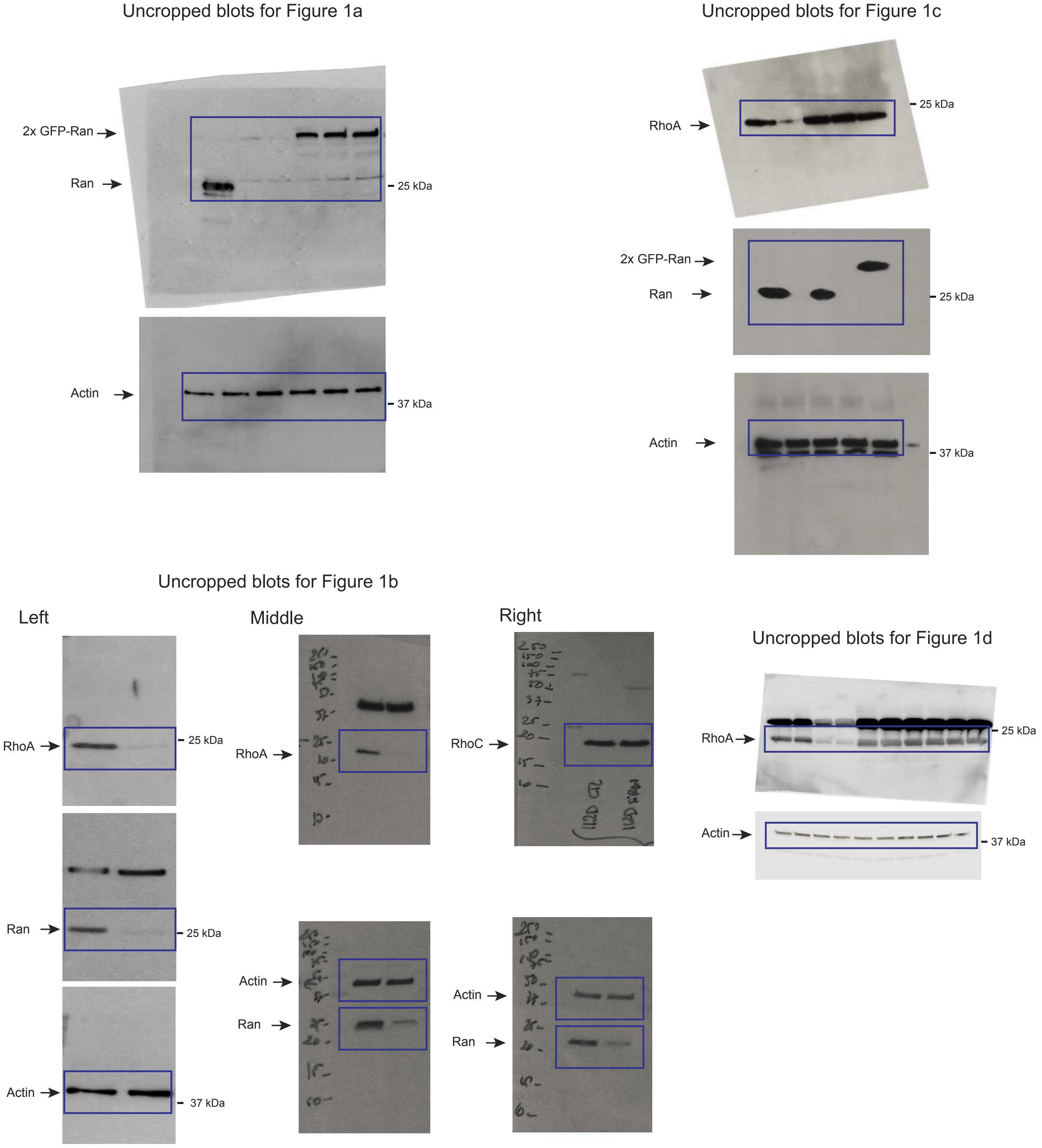

Supplementary Figure 4 Uncropped western blots. Blue boxes in the uncropped blots indicate the cropped regions shown in the corresponding figures.

Supplementary Figure 4 (continued)

Uncropped blots for Figure 1e

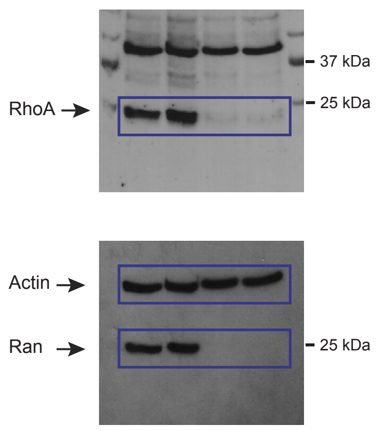

Uncropped blots for Figure 1f

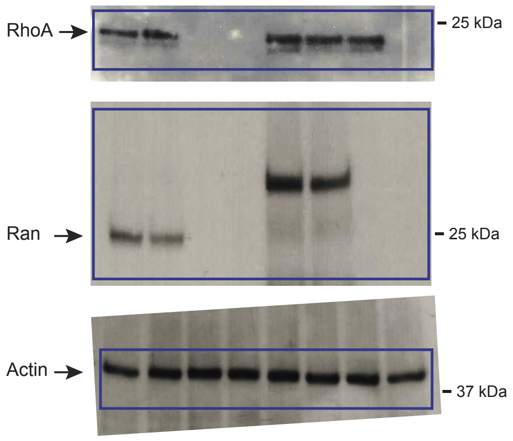

Uncropped blots for Figure 1h

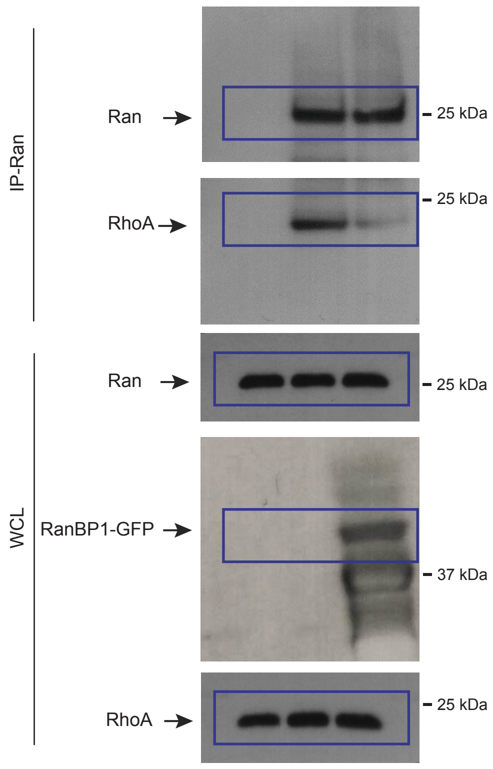

Uncropped blots for Figure 1i

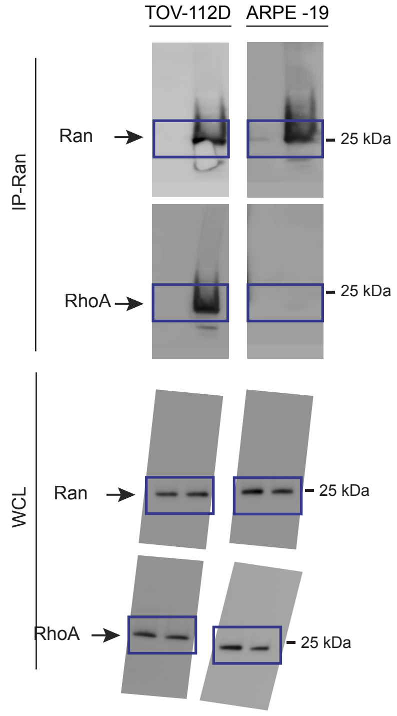

Supplementary Figure 4 (continued)

Uncropped blots for Figure 2c

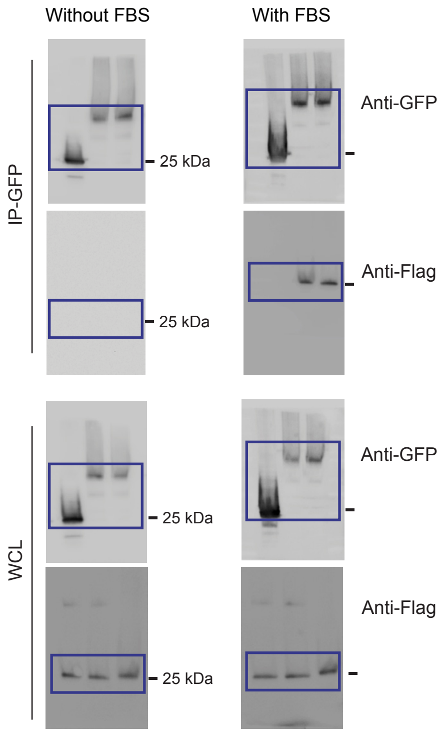

Uncropped blots for Figure 2d

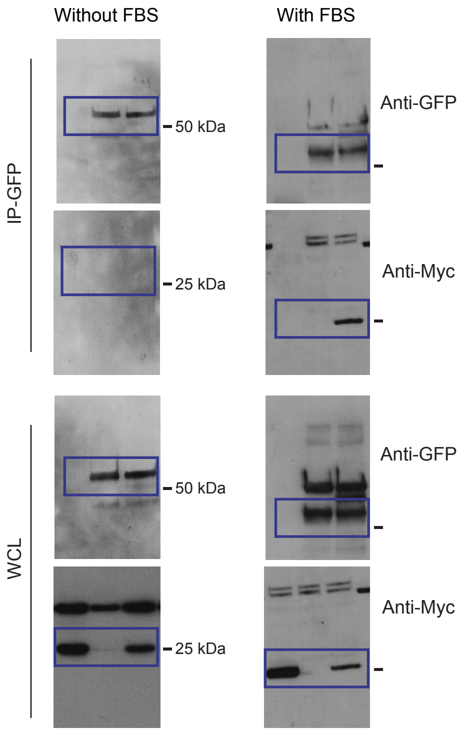

Uncropped blots for Figure 2e

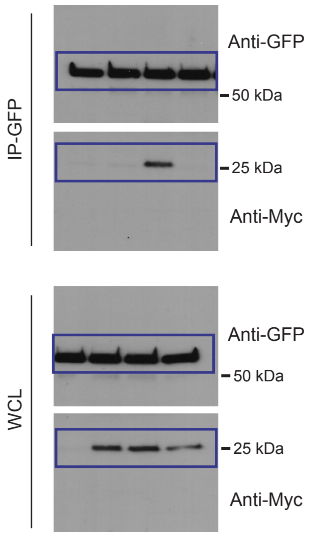

Uncropped blots for Figure 2f

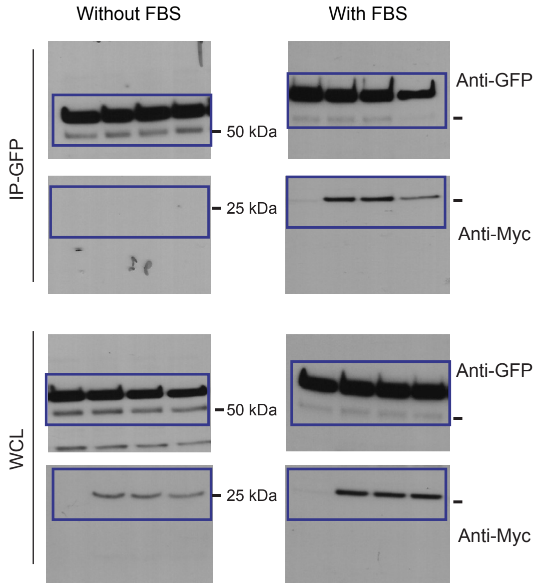

Uncropped blots for Figure 2g

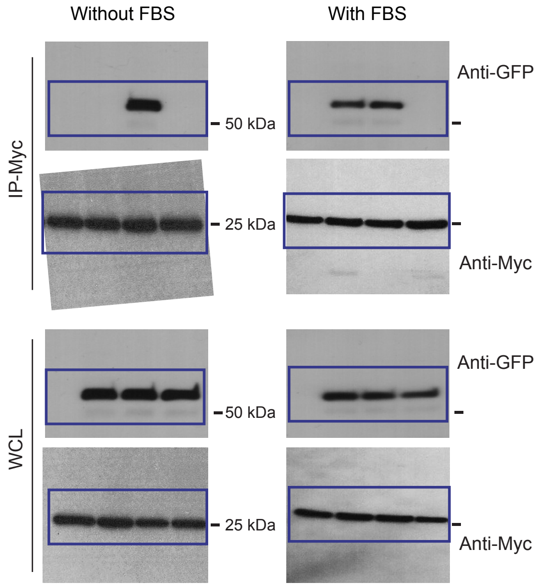

Supplementary Figure 4 (continued)

Uncropped blots for Figure 2h

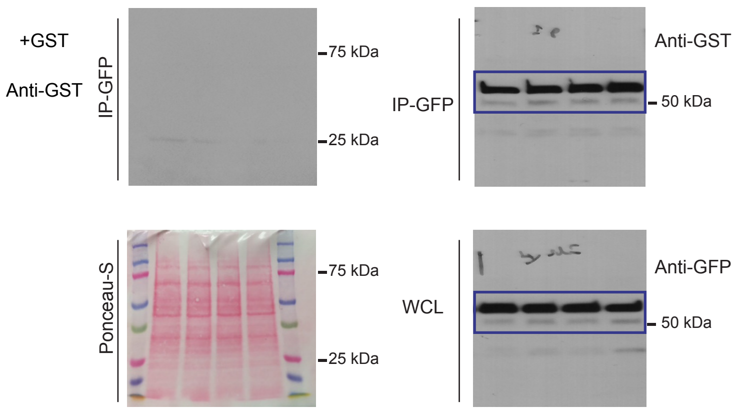

Uncropped blots for Figure 3b

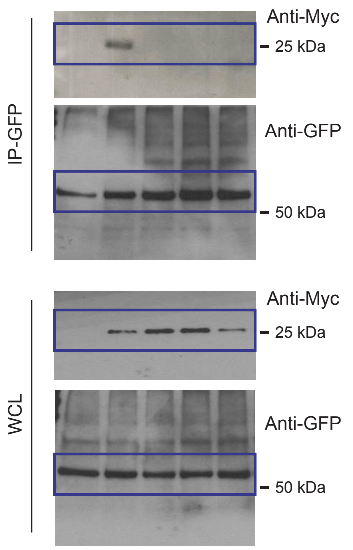

Uncropped blots for Figure 3c

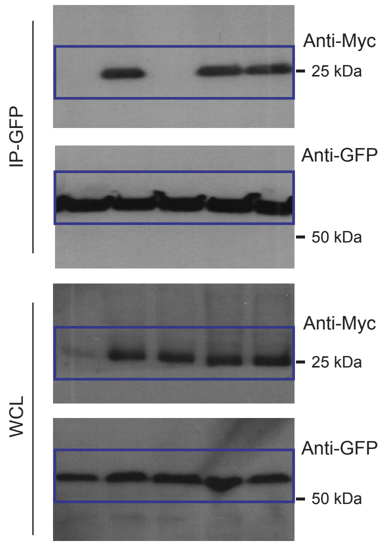

Uncropped blots for Figure 3d

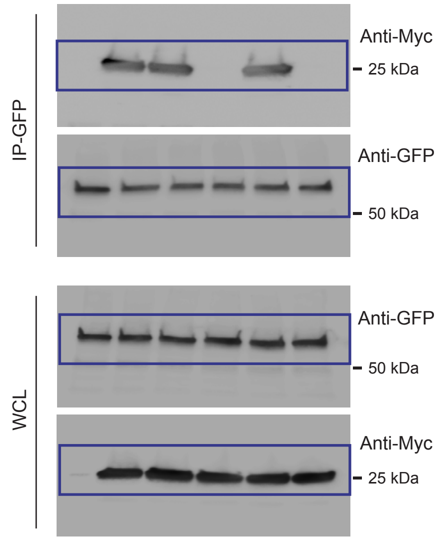

Uncropped blots for Figure 3e

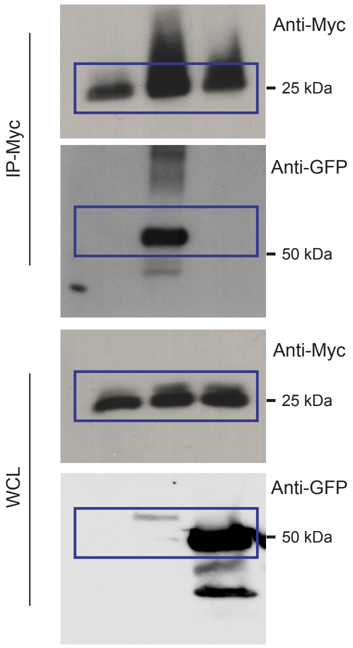

Supplementary Figure 4 (continued)

Uncropped blots for supplementary Figure 1a

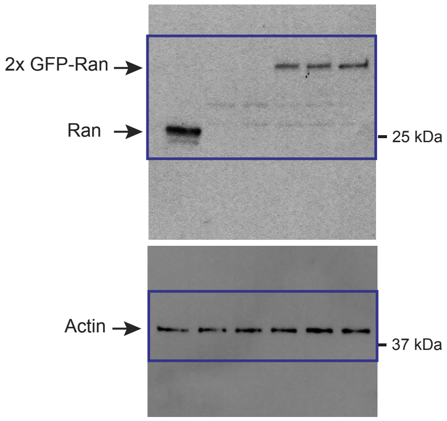

Uncropped blots for supplementary Figure 1d

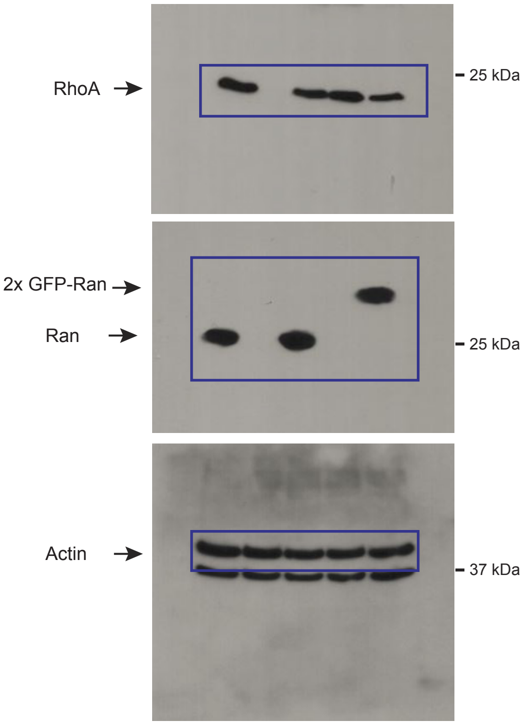

Uncropped blots for Supplementary Figure 1c

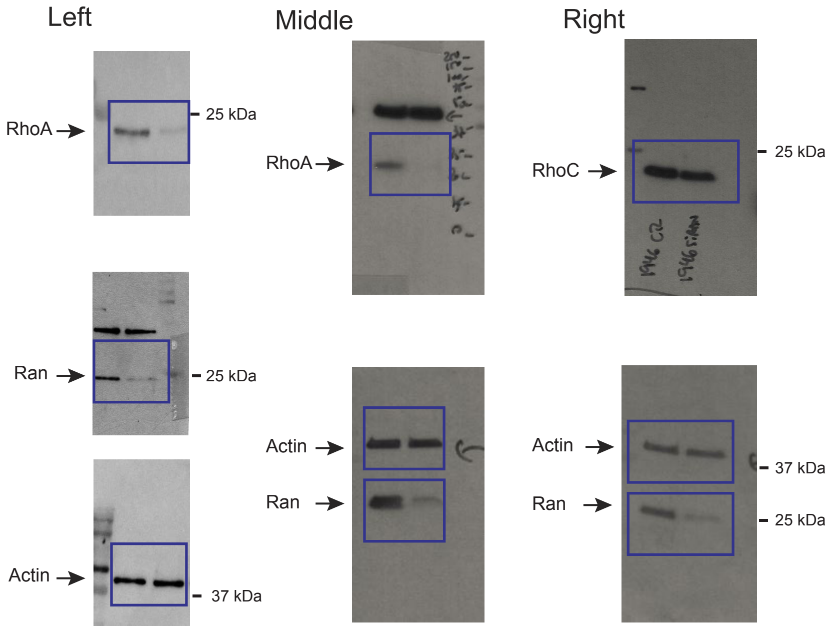

Uncropped blots for supplementary Figure 1f

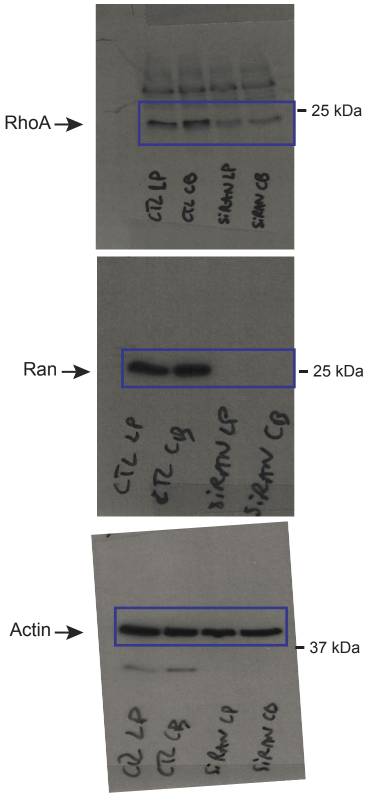

Supplementary Figure 4 (continued)

Uncropped blots for supplementary Figure 2c

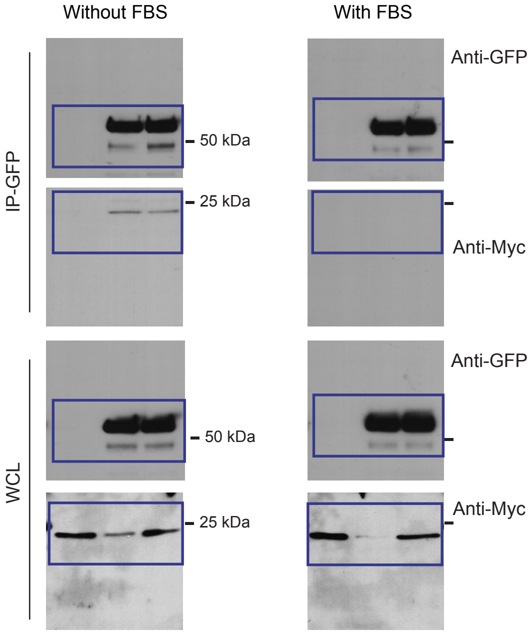

Uncropped blots for supplementary Figure 3c

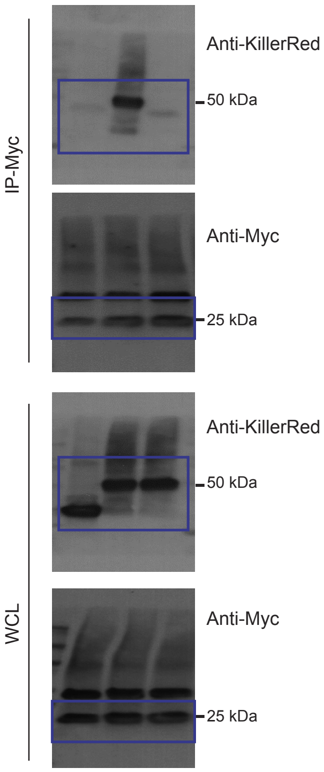

Supplement: Supplementary file 1 — Supplementary Information [file 41467_2019_10570_MOESM1_ESM.pdf]
