# Supplementary material for: Ran promotes membrane targeting and stabilization of RhoA to orchestrate ovarian cancer cell invasion
Source: Nat Commun. 2019 Jun 17;10:2666. doi: 10.1038/s41467-019-10570-w (PMC6573066; doi:10.1038/s41467-019-10570-w)
Supplement: Supplementary file 2 — Description of Additional Supplementary Files [file 41467_2019_10570_MOESM2_ESM.docx]

Description of Additional Supplementary Files

**Supplementary Movie 1:** Migration of TOV-112D cells after transfection with control siRNA

**Supplementary Movie 2:** Migration of TOV-112D cells after transfection with Ran siRNA
