# Supplementary material for: Ran promotes membrane targeting and stabilization of RhoA to orchestrate ovarian cancer cell invasion
Source: Nat Commun. 2019 Jun 17;10:2666. doi: 10.1038/s41467-019-10570-w (PMC6573066; doi:10.1038/s41467-019-10570-w)
Supplement: Supplementary file 6 — Reporting Summary [file 41467_2019_10570_MOESM6_ESM.pdf]

nature research

Corresponding author(s): DBPR NCOMMS-18-21431B

Last updated by author(s): Apr 25, 2019

Reporting Summary

Nature Research wishes to improve the reproducibility of the work that we publish. This form provides structure for consistency and transparency in reporting. For further information on Nature Research policies, see Authors & Referees and the Editorial Policy Checklist.

Statistics

For all statistical analyses, confirm that the following items are present in the figure legend, table legend, main text, or Methods section.

n/a	Confirmed	
☐	☒	The exact sample size (*n*) for each experimental group/condition, given as a discrete number and unit of measurement
☐	☒	A statement on whether measurements were taken from distinct samples or whether the same sample was measured repeatedly
☐	☒	The statistical test(s) used AND whether they are one- or two-sided<br>*Only common tests should be described solely by name; describe more complex techniques in the Methods section.*
☒	☐	A description of all covariates tested
☒	☐	A description of any assumptions or corrections, such as tests of normality and adjustment for multiple comparisons
☐	☒	A full description of the statistical parameters including central tendency (e.g. means) or other basic estimates (e.g. regression coefficient) AND variation (e.g. standard deviation) or associated estimates of uncertainty (e.g. confidence intervals)
☒	☐	For null hypothesis testing, the test statistic (e.g. *F*, *t*, *r*) with confidence intervals, effect sizes, degrees of freedom and *P* value noted<br>*Give P values as exact values whenever suitable.*
☒	☐	For Bayesian analysis, information on the choice of priors and Markov chain Monte Carlo settings
☒	☐	For hierarchical and complex designs, identification of the appropriate level for tests and full reporting of outcomes
☐	☒	Estimates of effect sizes (e.g. Cohen's *d*, Pearson's *r*), indicating how they were calculated

Our web collection on statistics for biologists contains articles on many of the points above.

Software and code

Policy information about availability of computer code

Data collection	For data collection: ZEN LE software 2 (Carl Zeiss®), AxioVision, Rel. 4.9.1 (Carl Zeiss®), EVOS FLc Cell Imaging System®, Versamax microplate reader at 490 nm (Molecular devices®), IncuCyte™ Zoom software
Data analysis	Data analysis was carried out with Microsoft Excel® 2010, Imaris software 8.2.0 (Bitplane®), IncuCyte™ Zoom software, Adobe Photoshop CC® using their standard functions/modules.

For manuscripts utilizing custom algorithms or software that are central to the research but not yet described in published literature, software must be made available to editors/reviewers. We strongly encourage code deposition in a community repository (e.g. GitHub). See the Nature Research guidelines for submitting code & software for further information.

Data

Policy information about availability of data

All manuscripts must include a data availability statement. This statement should provide the following information, where applicable:

- Accession codes, unique identifiers, or web links for publicly available datasets
- A list of figures that have associated raw data
- A description of any restrictions on data availability

The authors declare that the data supporting the findings of this study are available within the paper and its supplementary information files. If needed, additional information is available from the corresponding author upon reasonable request.

Field-specific reporting

Please select the one below that is the best fit for your research. If you are not sure, read the appropriate sections before making your selection.

☒ Life sciences ☐ Behavioural & social sciences ☐ Ecological, evolutionary & environmental sciences

For a reference copy of the document with all sections, see nature.com/documents/nr-reporting-summary-flat.pdf

Life sciences study design

All studies must disclose on these points even when the disclosure is negative.

Sample size	The chosen sample size are based on the numbers used for previous publications, which is most optimal to generate statistically significant results.
Data exclusions	No data were excluded for statistical analyses.
Replication	All attempts at replication were successful.
Randomization	Randomization was not relevant in this study as there was no comparison of cohorts.
Blinding	Blinding was not relevant to the study because all cells/samples were analyzed in the same way.

Reporting for specific materials, systems and methods

We require information from authors about some types of materials, experimental systems and methods used in many studies. Here, indicate whether each material, system or method listed is relevant to your study. If you are not sure if a list item applies to your research, read the appropriate section before selecting a response.

Materials & experimental systems

n/a	Involved in the study	
☐	☒	Antibodies
☐	☒	Eukaryotic cell lines
☒	☐	Palaeontology
☒	☐	Animals and other organisms
☒	☐	Human research participants
☒	☐	Clinical data

Methods

n/a	Involved in the study	
☒	☐	ChIP-seq
☒	☐	Flow cytometry
☒	☐	MRI-based neuroimaging

Antibodies

Antibodies used	anti-Ran (sc-271376) diluted 1:1000 (Santa Cruz Biotechnology), anti-RhoA (sc-418) diluted 1:100 (Santa Cruz Biotechnology), anti-RhoC (sc-393090) diluted 1:100 (Santa Cruz Biotechnology), anti-GST (sc-138) diluted 1:100 (Santa Cruz Biotechnology), anti-Myc (sc-764) diluted 1:100 (Santa Cruz Biotechnology), anti-RhoC (sc-393090) diluted 1:100 (Santa Cruz Biotechnology), anti-GST (sc-138) diluted 1:100 (Santa Cruz Biotechnology), anti-Myc (sc-764) diluted 1:100 (Santa Cruz Biotechnology), anti-GFP (11814460001) diluted 1:100 (Roche®), anti-Flag (F3165) diluted 1:100 (Sigma®), KillerRed (AB961), diluted 1:1000 (Evrogen®), anti-actin (ab6276), diluted 1:10000 (Abcam®), anti-mouse (sc-2061) diluted 1:10000 (Santa Cruz Biotechnology), anti-mouse (D3V2A) diluted 1:1000 (Cell Signaling Technology®). For Immunofluorescence : anti-RhoA (sc-179) diluted 1:50 (Santa Cruz Biotechnology), anti-Myc (sc-764) diluted 1:50 (Santa Cruz Biotechnology), Alexa Fluor 488 or 546 diluted 1:500 (Molecular Probes_Thermo Fisher Scientific®). For RhoA activity : anti-RhoA diluted 1:50 (Cytoskeleton®), HRP–conjugated secondary antibody diluted 1:250 (Cytoskeleton®)
Validation	Antibody validation was deferred to the manufacturers and was supported by multiple publications.

Eukaryotic cell lines

Policy information about cell lines

Cell line source(s)	ARPE-19 a human retinal pigment epithelial cell line was purchase from ATCC (#CRL2302). The TOV-112D and TOV-1946 ovarian cancer cell lines were respectively derived from a high-grade endometrioid tumor and a high-grade serous carcinoma, and were used to downregulate the expression of Ran and RhoA. Both cell lines are known to express high levels of Ran and have been previously described by our lab as indicated in Methods section.
Authentication	TOV-112D and TOV-1946 ovarian cancer cell lines were used with further authentication: Short Tandem Repeat (STR) analysis

Mycoplasma contamination	Cell lines were tested negative for mycoplasma contamination.
Commonly misidentified lines (See ICLAC register)	No commonly misidentified cell lines were used in this study.
